# AF among Nurses Working in Neonatal and Paediatric Intensive Care Units: A Cross-Sectional Study

**DOI:** 10.3390/healthcare12161574

**Published:** 2024-08-08

**Authors:** Taibah M. Ali, Manal F. Alharbi

**Affiliations:** 1 Maternal & Child Health Nursing Department, College of Nursing, King Saud University, Riyadh 12371, Saudi Arabia; 2 Ministry of Health, Heraa General Hospital, Makkah 24227, Saudi Arabia

**Keywords:** clinical alarms, fatigue, intensive care units, nurses, paediatrics

## Abstract

**Aim**: This research study aims to determine nurses’ alarm fatigue (AF) levels in paediatric critical care units in two governmental hospitals and to examine the significant differences in the mean between nurses’ attributes, nurses’ working environment, and nurses’ alarm management with the level of fatigue caused by the alarm. **Background**: In recent years, AF has become a significant and growing concern among nurses. However, in the Saudi Arabian paediatrics context, the impact of AF on nurses working in intensive care units remains unexplored. **Method**: A descriptive cross-sectional survey was conducted using a non-probability purposive sampling method. Data were collected from 216 nurses in two governmental hospitals through self-administered questionnaires comprised of four sections: individual attributes, work environment, alarm management, and AF scale. **Data analysis**: The Statistical Package of Social Science (SPSS) was used to analyse the data, and ANOVA was utilised to describe the sample’s demographic characteristics and determine any differences. **Results:** Most participants were female, held a bachelor’s degree, and were aged 31 to 35. Of the participants, 62.5% reported experiencing a medium level of AF, 29.2% reported a low level, and 8.3% reported a high level. Participants expressed that recurrent false alarms disrupt patient care and decrease trust in alarm systems. Significant differences in AF levels were observed based on marital status and the percentage of non-actionable alarms. **Conclusions**: Nurses working in paediatric critical units with high rates of false alarms, the frequent de-activation of alarms, and decreased trust in alarm systems are more likely to experience AF. Addressing AF is crucial for patient safety; nurse training on alarm management, the collaboration between biomedical and nursing staff, and technological advancements can help mitigate this issue. **Implications for Practice**: To minimise the adverse effects of AF, policymakers, biomedical experts, and nursing administrators must establish comprehensive policies and protocols concerning alarms. These measures aim to ensure secure and efficient care for the well-being of patients and nurses.

## 1. Introduction

Critical care units are specialised hospital departments that monitor and treat patients with multidisciplinary health needs. Nurses in paediatric intensive care units (PICUs) and neonatal intensive care units (NICUs) are crucial in providing comprehensive and specialised care for critically ill infants and children. These nurses are responsible for continuously monitoring patients’ vital signs, administering medications, and responding to changes in their condition [1]. Nurses monitor the condition of patients 24 h a day, leading to an average of 150 to 400 alarms per patient that they must respond to [2]. They must possess a deep understanding of the unique physiological and developmental needs of their young patients as well as the ability to quickly recognise and address any potential complications [1].

Alarm fatigue (AF), a phenomenon experienced by nurses in intensive care units, arises from repeated exposure to various alarm devices, each with different alerts. These alarms are designed to notify nurses of life-threatening conditions or changes in a patient’s situation [3]. Unfortunately, the excessive prevalence of false alarms in critical care units is a leading cause of AF, negatively impacting nurses’ performance and well-being [4]. Prolonged exposure to alarms induces stress in nurses, which can result in occupational burnout. Surprisingly, 85–99% of all alarms are clinically insignificant or false [2]. 

AF has been studied previously, for instance [5]. A 2020 systematic review found that many nurses required knowledge about AF, and high noise levels contributed to fatigue. Prolonged exposure to these factors can have adverse health effects on nurses and increase the risk of stress and burnout due to a lack of technological capability to address them [2]. Previous studies have examined AF, highlighting the potential benefits of implementing an alert management strategy. Such a strategy could reduce healthcare costs and save nurses valuable time, enabling them to focus more on providing direct patient care. For instance, a study conducted at the Geneva University Hospital in Switzerland analysed the prevalence and causes of infusion alarms in a paediatric intensive care setting. The study found a higher frequency of alarms compared to adult intensive care units (ICUs), with an average of 2164 alerts per hour from infusion pumps. Paediatric-specific factors such as infant agitation, movement, IV lines, infusions, and bolus injections contributed to these alarms [6]. 

A study conducted in Lebanon in 2020 involved nurses and physicians from various adult and paediatric critical care units [3]. The results showed that high levels of AF were linked to reports of stress, anxiety, or diminished quality of life. The study underscored the importance of implementing multidisciplinary approaches to mitigate AF, such as staff and equipment training, protocols, and guidelines. It is important to note that the study’s small sample size and use of convenience sampling may need revision to improve the generalisability of its findings. In a recent survey, a study [7] examined the impact of an alarm management initiative on reducing AF among critical and telemetry nurses. Utilising a quasi-experimental design, participants were divided into two groups. The control group received standard alarm management instructions, while the intervention group received specific guidance. The findings highlighted that implementing alarm management strategies helped diminish AF and enhanced the well-being of nurses [7]. In 2020, the Emergency Care Research Institute ECRI highlighted AF as the sixth-most dangerous technological hazard in healthcare facilities, impacting patient safety and resulting in high staff burnout due to the constant need to attend to alarms (2020). The consequences of AF included lower job satisfaction, increased stress and anxiety, and compromised patient safety [8]. In Saudi Arabia, the King Faisal Specialist Hospital and Research Centre in Jeddah, Saudi Arabia, created clinical decision support systems (CDSS) to tackle the issue of caregivers disregarding the large number of false alarms. A thorough online survey, influenced by existing literature and studies, suggested effective strategies to assist physicians in handling alarms and mitigating fatigue. Following a literature review and CDSS survey, researchers pinpointed five key strategies to combat AF, classifying alert types and messages into active and passive categories based on alert intensity [9]. Prioritising patient safety and adopting an alarm strategy can assist nurses in discerning between relevant and actionable alarms. The impact of AF on patient safety and care providers has been well-documented. AF can lead to performance deterioration, affecting the quality of patient care [10]. 

Additionally, continuous exposure to high sound levels can cause significant changes in infants’ physiology and behavioural reactions, potentially disrupting their average growth and development [11]. Loud noises prevent infants from sleeping, which is crucial for the growth of their central nervous systems [12]. Most NICU noise levels exceed American Academy of Paediatrics standards despite the potential risks. Noise levels exceeding 45 decibels may cause cochlear damage and disrupt premature infants’ average growth and development [13]. 

The first incident of AF was reported in 1974 by the Emergency Care Research Institute ECRI when a nurse ignored the alarm of a hypothermia machine, leading to a patient burn [4]. Therefore, hospitals must assess the risk of noise exposure and take steps to control the workplace’s noise levels to protect patient health and employee safety [14]. Based on previous studies and literature about AF, in 2023, Ali Al-Quraan discovered that oncology nurses are at a high risk of experiencing AF, leading to a call for interventions to address this issue [15]. However, there is a significant gap in the literature regarding measuring AF levels and identifying contributing factors, specifically among nurses in the paediatric context. Therefore, our study seeks to fill this gap by being the first to examine the level of AF and its contributing factors in the paediatric setting. By focusing on these specific critical units, our research aims to provide valuable insights into the unique challenges faced by paediatric nurses and contribute to developing effective interventions to mitigate AF in this context. This study examines AF among nurses within paediatric context units in two hospitals in Makkah. The main aim of the current research has been achieved through the following objectives:

1. To determine nurses’ AF levels in the paediatric critical care units in two hospitals in Makkah City.
2. To examine the significant differences in the mean between nurses’ attributes, nurses’ working environment, and nurses’ alarm management with the level of fatigue from the alarm.

## 2. Materials and Methods

### 2.1. Study Design and Setting

A cross-sectional descriptive approach was employed. By focusing on a specific healthcare setting (facility-based approach), we aimed to capture a snapshot of AF levels among nurses in the targeted units. This approach allowed us to gather valuable insights into the current state of AF in this setting. This study was conducted at two government hospitals in Makkah, Western Saudi Arabia. Hera General Hospital (HGH) has approximately 400 beds for adult and paediatric care and serves a diverse patient population. It provides specialised care for paediatric patients, including an NICU and a PICU. Maternity and Children’s Hospital (MCH) has a combined bed capacity of 621 beds in its ICU and emergency room. The hospital offers various paediatric medical subspecialties, including oncology, a clinical section for paediatric care, and a delivery room. Similarly, this hospital’s healthcare providers consist of Saudi and non-Saudi professionals who use state-of-the-art medical equipment. 

### 2.2. Sampling

The study utilised non-probability purposive sampling, which involves selecting participants based on specific criteria. For our research, we included nurses with experience in paediatric critical care units and those who encountered issues related to the sound of alarms. The study involved Saudi and non-Saudi nurses with various academic qualifications but excluded managerial personnel, head nurses, and those with limited training or in specific nursing roles. To ensure a certain level of confidence in selecting the participants and to account for potential errors, we used the Slovin formula (see Equation (1)) to determine the sample size. The Slovin formula is a commonly used method to calculate sample sizes in research studies. It is particularly applicable when using a non-probability sampling method, such as purposive sampling, as in our study. We required 216 nurses working in PICU and NICU units across two governmental tertiary hospitals. Our confidence level was 95%, and we allowed a margin of error (e) of 5%. The first hospital had a total capacity of 70 beds in the NICU and 20 beds in the PICU, with approximately 1000 patient admissions annually. The second hospital had a capacity of 40 NICU beds and a PICU bed capacity of 11, with an approximate annual admission rate of 600 patients. Both hospitals had modern machinery, including electronic beds, syringe pumps, blood warmers, cardiac monitors, and feeding machines. The study included all nurses in the PICU and NICU units in both hospitals, except those in managerial positions, nurses who had recently left the units, and nurses with less than three months’ experience.

Slovin formula

(1) $$n=\frac{N}{1+Ne^{2}}$$

Note. Slovin’s formula: *n* is the number of samples; *N* is the total population in the study (N); *e* is the error level in the sampling; source (sciencing.com). 

### 2.3. Instrument

In June 2022, the authors granted permission to use the AF tool created by [16]. The university’s ethical committee and the Ministry of Health cluster authorised data collection from the two hospitals’ NICU and PICU departments. Participants were recruited from selected settings based on specific criteria, and those who agreed to participate were provided with a Google Form. This form explained the purpose of the study, assured anonymity and confidentiality, and emphasised the voluntary nature of their participation. The participants were also given instructions on how to complete the survey and were provided with the researcher’s contact information. The survey included individual attributes, work environment, and alarm management questions. The authors developed the individual attribute questions to align with the study’s objectives, while the work environment and alarm management questions were adapted from another study [3]. The individual attributes section included questions regarding age, gender, marital status, education qualifications, job position, and overall experience. The work environment section assessed factors such as experience in the current unit and patient ratio. The alarm management section explored variables such as the rate of false alarms, nurses’ reactions to continuous alarms, the staff responsible for alarm setting, and trust in the alarm system. A questionnaire was developed to evaluate AF. AF was assessed using a questionnaire comprised of 13 statements addressing nurses’ reactions to alarm sounds during duty hours. Each statement was assigned a score on a scale of 0 to 4, except for questions 1 and 9. A score of 0 represented “always”, 1 represented “usually”, 2 represented “occasionally”, 3 represented “rarely”, and 4 represented “never” (except for questions 1 and 9, which had reversed scoring). If a nurse responded “never” to questions 1 or 9, the score was 4, indicating high impact from the alarm. Conversely, if the response was “always”, the score was 0, indicating a correct response.

The AF questionnaire was validated and proven reliable for measuring AF among nurses [16]. To evaluate the relevance and accuracy of the questionnaire’s items, the Content Validity Index (CVI) was used, involving a minimum of 3 experts, as recommended, but no more than 10 [17]. In this study, four academic researchers assessed the appropriateness of the questionnaire related to AF. The evaluations conducted by these experts resulted in a CVI of 1, establishing the questionnaire’s validity. They determined that no elements needed to be excluded or added to the scale. Internal homogeneity and retest methods were used to assess the reliability of the nursing AF questionnaire. The test–retest correlation coefficient was 0.99, the Guttman split-half correlation coefficient was 0.79, and Cronbach’s alpha was 0.91 [16]. For this study specifically, the AF questionnaire demonstrated favourable internal consistency, with a Cronbach’s alpha of 0.82. Additionally, most items exhibited item-total correlations above 0.3 and below 0.9, indicating a satisfactory level of discrimination.

### 2.4. Data Collection

The survey was administered online via Google Forms (https://www.google.com/forms/about/, Google LLC, Mountain View, CA, USA) and later exported to Microsoft Excel (Microsoft Office 10), with the data cleaned and transformed to SPSS version 26.0 (SPSS Inc., Chicago, IL, USA). The survey began with a cover letter explaining the purpose of the study; ensuring anonymity and confidentiality and highlighting the voluntary nature of participation, their right to withdraw at any time, and the lack of expected risk for participants; and reassurance that the data were being collected only for research purposes and that the data would be kept in a private folder accessed only by the researcher. The cover letter provided instructions on how to complete the survey, and participants were advised to contact the researcher if they had any questions or concerns. The use of an online survey allowed for easy distribution and completion. Including a cover letter ensured that participants were fully informed about the study and their rights as research participants. Each participant received a cover letter explaining the purpose of the study, the confidentiality of information, and the instructions to complete the questionnaire. These measures ensured the data collection process was organised and ethical and encouraged participation and cooperation. There are concerns regarding potential error rates associated with the utilisation of online Google Forms for data collection. To manage this, we conducted a pilot study to address instrument-related issues and ensure questionnaire clarity. Clear instructions were provided to participants, emphasising accuracy and data importance. Data validation techniques, such as pre-set response options, were implemented to minimise data entry errors. Regular communication and follow-up with participants helped address concerns and ensure data accuracy. While no method is error-free, these measures mitigated potential errors and ensured the reliability of the study’s findings. The link to the questionnaire was distributed to the participants using the QR code and sent to their department group via email to facilitate access to the questionnaire. In our study, we implemented several measures to ensure data completeness and minimise missing data. First, we designed the online questionnaire in such a way that the participants were required to respond to all essential variables. After collecting the data, we conducted a thorough data-cleaning process to review the responses for incompleteness or inconsistencies. We actively engaged with participants to address any missing data or seek clarification. These steps were taken to enhance the reliability and completeness of our data.

Data collection began on 5 March 2023 and lasted for three weeks until 27 March 2023.

### 2.5. Data Analysis

With no missing data, completed data helps us analyse and draw reliable conclusions. We used SPSS 26.0 to organise and examine the data. Our analysis included descriptive and inferential statistics. Descriptive statistics summarised each variable using frequency counts, percentages, standard deviations, and measures such as the mean. We used *t*-tests, ANOVA, and linear regression to predict the variables. Assumptions were met, with no collinearity or autocorrelation (Durbin–Watson = 1.682), and the residuals followed a normal distribution. We set a cut-off score on the AF Scale to better categorise the data and understand the research findings. Cut-off points help classify participants into different levels, such as—in this case—low, moderate, or high AF. Using cutoff points brings structure and standardisation to data analysis. These cutoff scores help in classifying data into different categories or levels, which can provide valuable insights and facilitate understanding of the research findings [18].

## 3. Results

The majority of participants were in the 31–35 age range (35.2%), followed by 26–30 years (22.7%) and 36–40 years (16.7%). A smaller proportion of participants fell into the age groups of 20–25 years (9.7%) and 41 years and above (15.7%). The vast majority, approximately 98.1%, were female. Out of the participants, roughly half, or 50.9%, were married and 86.1%, possessed a bachelor’s degree. Additionally, 97.7% identified themselves as staff nurses. However, 29.2% had spent one to two years in the NICU/PICU. For 62.0% of the cases, the patient-to-staff ratio was 1:2. In total, 41.7% of the alarms recorded were deemed non-actionable. A considerable number, approximately 65.7%, of participants chose to deactivate or mute persistent alerts. Furthermore, 76.9% had already implemented an alarm management policy. Notably, 77% were not solely responsible for setting alarm limits. For 56.0% of the participants, false alarms caused a reduction in trust. As shown in Table 1, the descriptive outcome indicates that the mean score for the AF level is 30.00, with a standard deviation of 7.35. Participants reported scores ranging from 11.00 to 48.00. Among the respondents, 29.2% experienced a low level of AF, while the majority, 62.5%, indicated a medium level, and a smaller group, 8.3%, reported a high level. These percentages reflect the valid proportions within each category. Table 1 presents the results for the AF statements.

### 3.1. Relationship between AF and Study Variables

Marital status has a notable impact on AF, as shown by a significant finding (F(2, 213) = 4.794, *p* = 0.009). Further analysis using the Tukey HSD test reveals a significant mean difference of −2.394 (*p* < 0.05) in AF levels between married and single nurses. However, no significant differences were observed concerning age groups, gender, nursing positions, years of experience, experience in the current unit, or patient ratios among nurses concerning fatigue levels.

The fatigue levels of nurses varied significantly based on the percentage of non-actionable alarms (false alarms), as indicated by the “Rate of Non-Actionable Alarms” variable. When the rate of non-actionable alarms was below 30%, nurses reported a mean fatigue level of 27.60 (SD = 7.45). However, this fatigue level increased significantly to 31.22 (SD = 6.55) when the rate ranged between 30% and 50% (F(1, 214) = 6.603, *p*-value < 0.001) and further rose to 35.36 (SD = 8.48) when the rate exceeded 70% of non-actionable alarms. The Tukey HSD test, used for post hoc analysis on the “Rate of Non-Actionable Alarms” variable, revealed significant differences and mean variations among the groups. No significant differences were observed for rates between 50% and 70% or rates beyond 70% of non-actionable alarms following post hoc tests. These findings highlight that fatigue levels differed significantly when the rate of non-actionable alarms was below 30% compared to rates between 30% and 50% (*p*-value < 0.001) as well as rates exceeding 70% (*p*-value < 0.001).

Regarding nurses’ responses to continuous alarms, those who answered “Yes” experienced a significantly higher mean fatigue level of 31.37 (SD = 7.38) compared to those who responded “No”, with a mean of 27.36 (SD = 6.56) (t(214) = 3.931, *p*-value < 0.001).

Nurses who indicated having a set of rules and procedures regarding alarms had a mean fatigue level of 29.63 (SD = 7.21), whereas those lacking such policies had a mean of 31.24 (SD = 7.74). Nonetheless, the distinction between the two groups was not statistically significant (t(214) = −1.364, *p* = 0.087). Nurses who were accountable for alarm limits showed a significantly higher mean fatigue level of 32.53 (SD = 7.84) compared to those who were not responsible, with a mean of 29.26 (SD = 7.05) (t(214) = 2.785, *p* = 0.006). Similarly, the factor “Loss of trust in alarms due to false alerts” indicated that nurses who concurred with this statement reported a significantly higher mean fatigue level of 32.34 (SD = 7.23) than those who disagreed, with a mean of 27.02 (SD = 6.38) (t(214) = 5.647, *p* < 0.001). Additionally, the nurses’ reactions to continuous alarms and trust (“Yes” vs. “No”) were found to be significantly different (*p* < 0.001).

### 3.2. Factors Contributing to AF

The study examined individual attributes, work environment, and alarm management variables with AF variables. Three predictor variables contributed to the variance in AF, with an R-square value of 0.223 and an adjusted R-square value of 0.205 (indicating slight overfitting). The model showed significance, as noted in the F-statistic (F(5, 210) = 12.078, *p* < 0.001). Non-actionable alarm frequency, deactivating or silencing continuous alarms, and reduced trust resulting from false alarms were found to predict AF. Specifically, a one-unit increase in non-actionable alarms led to a 1.632-unit increase in AF, deactivating or silencing alarms resulted in a 2.566-unit increase in AF, and reduced trust contributed to a 4.143-unit increase in AF (Table 2 and Table 3). 

## 4. Discussion

In this study, we conducted a quantitative analysis to examine the phenomenon of AF among nurses in neonatal and paediatric intensive care units. Our findings indicate that age does not significantly correlate with AF. This finding is consistent with another study [3], which found no significant relationship between age and nurse AF. However, previous studies have shown that age can be a substantial factor in developing AF. For instance, a strong association between age groups and levels of AF was identified, suggesting that senior nurses experience less AF than junior employees due to their higher levels of experience, knowledge, and skills [19].

However, the studies indicate no significant relationship between gender and AF [15,20]. It is worth noting that most nurses working in the PICU are female, and various physiological and psychological mechanisms influence how our brains process and react to sensory information. These mechanisms are influenced by genetics, lifestyle, and environmental factors, collectively contributing to individual variations in behaviour.

Our study found no significant connection between educational qualifications and AF. Our results align with the findings of [15], which used the same scale to assess AF in oncology nurses. Regarding nurses’ experience, we observed no notable differences among the four experience groups. Some researchers suggest that nurses with more extensive work experience may be more prone to experiencing AF compared to those with less experience [21]. This could be due to variations in individual experience levels, job responsibilities, and the ability to respond to alarms and emergencies based on critical thinking.

Most participants held bachelor’s degrees. Nursing education significantly influences nursing practice, problem-solving, professional growth, patient outcomes, and decision-making opportunities. The literature shows varying findings on individual attributes such as age, gender, educational qualification, and experience concerning AF. Our study consistently revealed no significant impact of work environment data on nurses’ fatigue levels. Similarly, the patient ratio did not affect nurses’ AF levels. While our study did not establish a significant link between work environment and AF, it highlights the importance of further research in understanding this relationship.

Various factors, such as heavy workload, understaffing, past negative experiences, knowledge and skills, and limited technological proficiency, greatly influence the management of alarms. AF poses a significant challenge in ICUs, as indicated by a previous study [21]. The study discovered that, on average, ICUs receive 43 alarms per hour, with 49.8% of these alarms considered clinically relevant at a medium or high level. However, only 37% of the alarms are responded to within 60 s, while 42.5% go unanswered. This suggests that AF leads to considerable delays in addressing clinically meaningful alarms, potentially affecting patient safety. Concerning alarm policies and procedures, nurses who acknowledged having such protocols experienced a higher level of fatigue than those who reported the absence of such protocols. However, this difference between the two groups did not reach statistical significance.

Moreover, several studies in the field advocate for implementing effective policies and procedures to address AF. Our study did not identify any specific alarm type as being significantly responsible for limitations and AF levels. Additionally, holding individuals accountable for adhering to alarm limits and perceiving false alarms as eroding trust in the alarm system contributes to AF. Concerning alarm policies and procedures, we contacted the head nurse of the selected departments, who confirmed the absence of such protocols. Furthermore, it was observed that only one facility, as disclosed by the director of the critical paediatric unit at MOH (personal communication, 16 May 2023), utilises sound level meters. Sound level meters are devices used to monitor and control noise levels in the NICU. By measuring noise levels in this unit, staff can identify areas with excessively high noise and implement measures to reduce it, such as adjusting equipment settings, using sound-absorbing materials, or limiting staff conversations.

According to the previous study [2], evaluating the extent of AF is vital for ensuring the well-being of patients and nursing staff and testing the efficacy of implemented strategies. Noise affects nurses in the critical paediatric unit in various ways, such as heightening stress levels, disrupting sleep, burdening their mental load, and creating distractions that impact their nursing performance. This is supported by the studies conducted by [2,3,4,10]. Nurses who experience high levels of AF are at a greater risk of stress and other related consequences. Several factors could contribute to those categorised as having moderate AF, including their extensive experience, job description, shift tasks, individual traits, and the effects of AF on their ability to adapt and cope with the unit’s demands.

Conversely, nursing staff may need to be aware of the consequences of not adjusting and setting alarm parameters according to the patient’s needs or the most effective practices. The rate of non-actionable alarms significantly impacts fatigue levels, with fatigue increasing when the percentage of false alarms rises from 30% to 70%, as observed by [3,4], indicating that false alarms also lead to AF. Moreover, according to another study [22], 72% of alerts within critical units are false, contributing to AF. Nurses can mitigate the number of false and non-actionable alerts by customising alarm settings, as demonstrated by [23] and the fact that nurses frequently encounter non-actionable alarms [24], which they believe cause delays in patient care. Multiple factors are associated with fatigue, including the rate of non-actionable alarms and nurses’ responses to continuous alarms.

The design of NICUs and PICUs is crucial for safeguarding the well-being of admitted children. A critical aspect of the department’s design is the integration of the alert and sound systems. These systems act as a link between patients and healthcare providers, catering to their various needs. Consequently, the unit’s design should prioritise the effective utilisation of sound. This involves thoughtfully selecting and strategically placing alarms within the unit, ensuring that only critical and urgent alarms reach the healthcare team while minimising false alarms. By doing so, healthcare professionals can focus solely on providing timely and accurate responses to alarms without compromising patient safety. The study revealed that nurses’ fatigue levels are linked to their responses to continuous alarms. Higher fatigue leads to the deactivation or altering of parameters to reduce alarms, as documented by [3,25], which align with our findings. Trust in alarms affects fatigue levels. A previous study [26] found a positive relationship between nurses’ trust in alarms and alarm accuracy. Factors for effective alarm management and patient safety include alarm reliability, appropriate settings, and education/training. Nurses accountable for alarm limits have higher fatigue levels. Another previous study [3] reported lower AF scores for clinicians who believed nurses are responsible for alert limit settings. The inability to adjust the alarm threshold leads to severe alarms. In addition, burnout is associated with noise within intensive care wards [27], which poses various risks to patients, including harm to their hearing and interference with the normal development of premature babies [11,28].

Specific solutions to AF can be adopted in the PICU; these solutions include adjusting alarm settings to individual patient’s needs, optimising alarm parameters to reduce false alarms, implementing a tiered alarm system for prioritisation, educating and training healthcare providers, monitoring and reporting AF, encouraging interdisciplinary collaboration, and evaluating and upgrading alarm technologies. By applying these solutions, healthcare teams can improve alarm system efficacy, minimise unnecessary alarms, increase response efficiency, and improve patient safety in the PICU.

## 5. Conclusions

This study in Saudi Arabia is the first to assess the impact of alarm fatigue (AF) and its contributing factors among nurses in governmental hospitals in Makkah City. Nurses stationed in units with frequent false alarms often resort to disabling alarms, lose confidence due to repeated false alarms, and are more likely to experience AF. AF sets in when nurses become desensitised to the overwhelming number of false or irrelevant alarms. This desensitisation can lead to longer response times or even missing essential alarms, resulting in a lack of awareness, unconscious neglect, or inappropriate alarm responses. Effective measures should be implemented to reduce unnecessary alarms, discourage frequent deactivation, and restore nurses’ trust in alarm systems. It is crucial to find ways to minimise errors that can significantly impact patients’ health and the overall environment of the ICU. Future studies may consider using a qualitative design to allow participants to express their feelings about the phenomenon more effectively and to test the instruments’ psychometric properties.

## 6. Patents

To ensure patient safety, it is essential to implement strategies aimed at tackling AF. Providing nurses with training and education on alarm management and prioritisation can assist in mitigating AF. Collaboration between biomedical and nursing staff is needed to keep up with the latest updates using those devices and know how to adjust them to meet patients’ needs. Furthermore, incorporating technological advancements and enhancing alarm algorithms can reduce AF.

## 7. Limitations of the Study

This research followed a cross-sectional descriptive design, so the outcome cannot be used to establish a cause-and-effect relationship between the variables under study. The study was limited to the Ministry of Health (MOH) sector within Saudi Arabia, restricting the applicability of the findings to this specific context. The study focused solely on critical paediatric nurses, overlooking other healthcare practitioners in the critical care environment. There is a potential for sampling bias due to the participant selection criteria. Future research could benefit from larger sample sizes across multiple hospitals and healthcare providers and a longitudinal design to gather responses over time.

## Figures and Tables

**Table 1:** Descriptive Analysis of AF (n = 216).

Variable		Frequency	Percentage	Mean (SD)	Maximum–Minimum
1—I regularly readjust the limits of alarms based on the clinical symptoms of patients.	Never	14	6.5%		
	Rarely	20	9.3%		
	Occasionally	40	18.5%		
	Usually	67	31.0%		
	Always	75	34.7%		
2—I turn off the alarms at the beginning of every shift.	Never	15	6.9%		
	Rarely	15	6.9%		
	Occasionally	20	9.3%		
	Usually	18	8.3%		
	Always	148	68.5%		
3—Generally, I hear a certain amount of noise in the ward.	Never	5	2.3%		
	Rarely	25	11.6%		
	Occasionally	55	25.5%		
	Usually	71	32.9%		
	Always	60	27.8%		
4—I believe much of the noise in the ward is from the alarms of the monitoring equipment.	Never	4	1.9%		
	Rarely	17	7.9%		
	Occasionally	38	17.6%		
	Usually	86	39.8%		
	Always	71	32.9%		
5—I pay more attention to the alarms in certain shifts.	Never	4	1.9%		
	Rarely	12	5.6%		
	Occasionally	31	14.4%		
	Usually	62	28.7%		
	Always	107	49.5%		
6—In some shifts the heavy workload in the ward prevents my quick response to alarms.	Never	21	9.7%		
	Rarely	31	14.4%		
	Occasionally	55	25.5%		
	Usually	68	31.5%		
	Always	41	19.0%		
7—When alarms go off repeatedly, I become indifferent to them.	Never	22	10.2%		
	Rarely	45	20.8%		
	Occasionally	77	35.6%		
	Usually	55	25.5%		
	Always	17	7.9%		
8—Alarm sounds make me nervous.	Never	22	10.2%		
	Rarely	35	16.2%		
	Occasionally	57	26.4%		
	Usually	54	25.0%		
	Always	48	22.2%		
9—I react differently to the low-volume (yellow) and high-volume (red) alarms of the ventilator.	Never	74	34.3%		
	Rarely	77	35.6%		
	Occasionally	40	18.5%		
	Usually	16	7.4%		
	Always	9	4.2%		
10—When I’m upset and nervous, I’m more responsive to alarm sounds.	Never	18	8.3%		
	Rarely	35	16.2%		
	Occasionally	65	30.1%		
	Usually	49	22.7%		
	Always	49	22.7%		
11—When alarms go off repeatedly and continuously, I lose my patience.	Never	41	19.0%		
	Rarely	62	28.7%		
	Occasionally	51	23.6%		
	Usually	31	14.4%		
	Always	31	14.4%		
12—Alarm sounds prevent me from focusing on my professional duties.	Never	38	17.6%		
	Rarely	44	20.4%		
	Occasionally	52	24.1%		
	Usually	44	20.4%		
	Always	38	17.6%		
13—During visiting hours, I pay less attention to the alarms of the equipment.	Never	87	40.3%		
	Rarely	52	24.1%		
	Occasionally	36	16.7%		
	Usually	27	12.5%		
	Always	14	6.5%		
AF				30.00 (7.35)	48.00/11.00
	Low	63	29.2%		
	Medium	135	62.5%		
	High	18	8.3%		

**Table 2:** Multiple linear regression analysis (n = 216).

Model	B	SE B	Beta Β	T	Sig.	95.0% CL for B		R Square	Adjusted R Square
						LL	UL		
(Constant)	38.111	3.214		11.859	<0.001	7.311	17.437	0.223	0.205
Marital status	1.073	0.854	0.077	1.256	0.210	−0.611	2.757		
Rate of (false alarms)	1.632	0.545	0.188	2.995	0.003	0.558	2.707		
Deactivation or Silencing the Alarms	2.566	0.974	0.166	2.635	0.009	0.646	4.486		
responsible for alarm limit setting	1.870	1.086	0.107	1.722	0.086	−0.270	4.011		
your trust in alarm systems	4.143	0.932	0.281	4.444	<0.001	2.305	5.981		

**Table 3:** Analysis of variance (ANOVA) for multiple regression model (n = 216).

Model	Sum of Squares	Df	Mean Square	F	Sig.
Regression	3001.023	13	230.848	5.420	<0.001
Residual	8602.977	202	42.589		
Total	11,604.000	215			

## Data Availability

The data are available upon reasonable request from the corresponding author.
